# Modeling the influence of short term depression in vesicle release and stochastic calcium channel gating on auditory nerve spontaneous firing statistics

**DOI:** 10.3389/fncom.2014.00163

**Published:** 2014-12-23

**Authors:** Bahar Moezzi, Nicolangelo Iannella, Mark D. McDonnell

**Affiliations:** Computational and Theoretical Neuroscience Laboratory, Institute for Telecommunications Research, University of South AustraliaMawson Lakes, SA, Australia

**Keywords:** calcium dynamics, stochastic synapse, inner hair cell, auditory nerve, short term depression, neural variability, channel noise

## Abstract

We propose several modifications to an existing computational model of stochastic vesicle release in inner hair cell ribbon synapses, with the aim of producing simulated auditory nerve fiber spiking data that more closely matches empirical data. Specifically, we studied the inter-spike-interval (ISI) distribution, and long and short term ISI correlations in spontaneous spiking in post-synaptic auditory nerve fibers. We introduced short term plasticity to the pre-synaptic release probability, in a manner analogous to standard stochastic models of cortical short term synaptic depression. This modification resulted in a similar distribution of vesicle release intervals to that estimated from empirical data. We also introduced a biophysical stochastic model of calcium channel opening and closing, but showed that this model is insufficient for generating a match with empirically observed spike correlations. However, by combining a phenomenological model of channel noise and our short term depression model, we generated short and long term correlations in auditory nerve spontaneous activity that qualitatively match empirical data.

## 1. Introduction

In the vertebrate auditory pathway, the inner hair cell and auditory nerve (IHC-AN) complex is the principal structure for the transduction of basilar membrane motion to stochastic trains of action potentials (Mulroy et al., 1974; Glowatzki and Fuchs, 2002; Johnson et al., 2009; Matthews and Fuchs, 2010). A computational model of the IHC-AN complex was proposed by Meddis (1986), and later modified by Sumner et al. (2002) to become a component in a larger computational model of the transformations of sounds by the middle ear. Unlike the Meddis (1986) model, in the Sumner et al. (2002) model, vesicle release from the IHC to the cleft was conceptualized as quantal and accruing with a probability that had a third power dependence on pre-synaptic calcium concentration. Later, the Sumner et al. (2002) model was modified by Meddis (2006) to take into account more physiological functions.

Here, we present a revised version of the Meddis (2006) model of the IHC-AN complex, with the aim of enhancing understanding of the biophysical sources of stochastic variability in the IHC-AN complex, by generating auditory nerve spontaneous spiking that provides an improved statistical match with empirical data.

The Meddis (2006) model includes a probabilistic “relative refractoriness” component, which is designed to replicate observed variation in the minimum time between spikes in AN fibers. Here we propose a pre-synaptic physiological explanation as the cause for what is attributed to post-synaptic relative refractoriness (note that we do not alter the original model's “absolute refractory” period, which models spike generation and membrane potential recovery). Specifically, we introduce a model of short term depression in pre-synaptic vesicle release, similar to short term plasticity models developed for cortical synapses (Tsodyks and Markram, 1997; Scott et al., 2012; Hennig, 2013; McDonnell et al., 2013). Unlike most such models, the conceptual model here is that there is a temporarily reduced probability of pre-synaptic vesicle release, following each actual release. Also unlike those models, the input to the synapse is not discrete spiking events, but instead the continuously valued membrane potential of the inner hair cell. The reason our model is suitable for capturing phenomena that have traditionally been attributed to post-synaptic relative refractoriness is that it introduces variability in the time between vesicle releases, which in turn leads to variability in the minimum time between post synaptic spikes. Our reasons for seeking this alternative conceptual model are given in the Discussion section.

We compare the resulting auditory nerve spontaneous firing statistics of our model with the firing statistics published by Heil et al. (2007). For spontaneous neural activity in auditory nerve fibers, inter-spike interval (ISI) distributions have been shown by Heil et al. (2007) to match empirical data better if the vesicle release inter-event interval (IEI) distribution was assumed to be a mixture of an exponential function and a gamma function with shape factor 2, both having the same scale parameters. We show that the probability density function (PDF) of ISI data obtained by Heil et al. (2007) fits PDF of ISI data obtained from our simulation if the time constant of short term depression is assumed to be around 2.5 ms.

Short and long term correlations have been observed in the spontaneous activity of auditory nerves (Teich, 1989; Lowen and Teich, 1992; Teich and Lowen, 1994). For individual auditory nerve fibers, it was shown that the Fano factor for spike counts increases for time scales from around 100 ms to tens of seconds indicating positive long term correlation and decreases slightly for time scales of around tens of milliseconds indicating short term negative correlation (Teich, 1989; Lowen and Teich, 1992; Teich and Lowen, 1994). Here we include a calcium channel noise model in the Meddis (2006) model. We show that for spontaneous activity, this biophysical noise model does not generate the short and long term correlations observed in the Teich and Lowen (1994) Fano factor curves.

However, we also modify the Meddis (2006) model to include a combination of a phenomenological model of IHC calcium channel noise and our model of short term depression in vesicle release. Using this model, for auditory nerve spontaneous activity, we generate Fano factor time curves that qualitatively match empirical Fano factor time curves of Teich and Lowen (1994); Teich (1989); Lowen and Teich (1992).

## 2. Materials and methods

Firstly, in Section 2.1, we review the previous models that our research is built upon:

- The inner hair cell model of Meddis (2006).
- The deterministic, stochastic and phenomenological synapse models of Meddis (1986), Meddis (2006), and Zilany et al. (2014).
- The vesicle-release-to-AN-spike-conversion models of Meddis (1986), Meddis (2006), Sumner et al. (2002) and Zilany et al. (2014).

Then in Section 2.2, we provide a review of previous statistical analysis of empirical auditory nerve spontaneous activity data including research published by Heil et al. (2007), Teich and Lowen (1994), Teich (1989) and Lowen and Teich (1992). The final models we describe in Section 2.3 are our modifications to the Meddis (2006) model. These are designed to enhance understanding of the biophysical origin of stochastic variability in AN spiking, and to generate auditory nerve spontaneous spiking that provides an improved statistical match with empirical results, as described in Section 2.2.

### 2.1. Previous models

#### 2.1.1. Inner hair cell model

Meddis (2006) describes a deterministic calcium-dependent model for converting the membrane potential of an inner hair cell, *v*(*t*), to a vesicle release rate, *k*(*t*). We use *c*(*t*) to describe the intra-cellular calcium concentration (relative to its rest concentration) as a function of time. In the model, the release-rate for available vesicles, *k*(*t*), is proportional to the cube of *c*(*t*). The calcium concentration depends on four constants, τ_*c*_, *G*_*c*_, *E*_*c*_, ν, on the membrane potential, *v*(*t*), and on an additional variable, *m*(*t*), where *m*^3^(*t*) represents the fraction of open channels at time *t* as well as the probability of a calcium channel to be open. This depends on three constants, γ, β and τ_*m*_, and on *v*(*t*). Note that *m*^3^(*t*) is bounded to the interval [0, 1], which is essential for it to physically represent a fraction of open channels. The maximum value of 1 occurs when *v*(*t*) is large and positive and the minimum value of 0 occurs when *v*(*t*) is large and negative.

In summary, the model has the following parameters:

- *b* is a parameter that can be varied to match data.
- *E_c_* is the calcium reversal potential.
- *G*_*c*_ is the maximum calcium conductance.
- τ_*c*_ is the time constant of calcium clearance.
- τ_*m*_, γ and β are constants that describe the voltage-dependent calcium current flow.
- ν is the unit correction constant.

The values of these parameters are summarized in Table 1. The equations describing conversion from *v*(*t*) to *k*(*t*) are

(1) $$k(t)\;=\text{max}(0,bc^{3}(t)),$$

(2) $$\frac{dc(t)}{dt}\;=-\frac{c(t)}{\tau_{c}}+\nu G_{c}m^{3}(t)(E_{c}-v(t)),$$

(3) $$\frac{dm(t)}{dt}\;=-\frac{m(t)}{\tau_{m}}+\frac{1}{\tau_{m}\left(1+\frac{e^{-\gamma v(t)}}{\beta }\right)},$$

where *k*(*t*) has units of releases per second. We have modified the Meddis (2006) and Sumner et al. (2002) models by introducing a constant ν with units of MA^−1^s^−1^ to ensure all terms in Equation (2) have units of Ms^−1^, where M is the unit of molar concentration. By fitting to the saccular hair cells of the bull-frog data, it has been shown (Hudspeth and Lewis, 1988) that

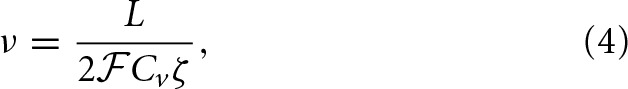



where 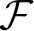 is Faraday constant, *C*_*v*_ is the cell volume, ζ is the fraction of cell volume where calcium is accumulated to and *L* is the proportion of free calcium in the neuron. The values of these parameters are summarized in Table 2, with the result that ν = 2.3× 10^9^ MA^−1^s^−1^.

**Table 1:** **Parameters for inner hair cell calcium levels**.

**Parameter**	**Description**	**Value**
*E*_*c*_ (*V*)	Calcium reversal potential	0.066
*G*_*c*_ (*S*)	Maximum calcium conductance	1.4 × 10^−8^
τ_*c*_ (*s*)	Calcium clearance time constant	240 × 10^−6^
τ_*m*_ (*s*)	Time constant of calcium current	5 × 10^−5^
γ (*V*^−1^)		100
β		400
*g*_*c*_ (*S*)	Single calcium conductance	15 × 10^−12^
*v* (*V*)	Intracellular inner hair cell potential	−0.0605

*The value of *g*_*c*_ was obtained from (Zampini et al., 2013). The value of *v* was obtained by running MAP__BS with no stimulus present. All other values are identical to those used in publicly available Matlab source code MAP__BS at http://www.essexpsychology.macmate.me/HearingLab/modelling.html*.

**Table 2:** **Parameters for calculating 
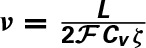
**.

**ν (MA^−^1s^−1^)**	***L***	**ζ (pl)**	***C*_*v*_**
2.3 × 10^9^	0.02	3.4 × 10^−5^	1.25

*Values obtained from Hudspeth and Lewis (1988)*.

To confirm that our proposed model enhancements have no effect on previously established model features, in the Results section we compare the average vesicle release rates obtained from simulation of the proposed model to the average vesicle release rate obtained from simulation of the Meddis (2006) model. We introduce the notation *k* as the simulated average vesicle release rate. We show that the changes that we make to Meddis (2006) model result in *k* that are close to *k* obtained from the original model of Meddis (2006). The parameter *k* for the various proposed models are summarized in the tables.

A positive calcium current is required to increase the calcium concentration but in the Meddis (2006) and Sumner et al. (2002) models, calcium current is negative (i.e., inward) when *v*(*t*) < *E*_*c*_. Therefore, we have used (*E*_*c*_ − *v*(*t*)) in Equation (2) instead of (*v*(*t*) − *E*_*c*_) used in the (Sumner et al., 2002) and (Meddis, 2006) models. The max(·) function is included in Equation (1) since although it is possible for *c*(*t*) < 0 in the model (which represents calcium concentration less than its rest value), the rate *k*(*t*) cannot be negative. Note that the final term in Equation (2) has the form of the deterministic (Hodgkin and Huxley, 1952) voltage-gated ion channel current model. Later, we replace this with a model of stochastically opening and closing ion channels.

#### 2.1.2. Deterministic synapse model

The input to the deterministic synapse model of Meddis (1986) is the rate at which the neurotransmitter is released to the cleft, *k*(*t*). There are three continuous-time-dependent variables that describe transport between a vesicle “factory,” an “immediate store,” the synaptic cleft, and a vesicle “recycling pool”:

- the amount of releasable neurotransmitter, *x*(*t*) ∈ [0, *M*]; where *M* is the maximum amount of neurotransmitter in the immediate store.
- the amount of neurotransmitter in the cleft, *y*(*t*).
- the amount of neurotransmitter being recycled, *z*(*t*).

There are four parameters that have units of rate:

- *r*_1_ is the rate of manufacture of neurotransmitter from the “factory.”
- *r*_2_ is the rate of restoration of neurotransmitter from the recycling pool.
- *r*_3_ is the rate at which neurotransmitter is lost in the cleft.
- *r*_4_ is the rate at which neurotransmitter is moved from the cleft to the recycling pool.

The values of these parameters are summarized in Table 3. The deterministic Meddis (1986) synapse model is of the following form

(5) $$\frac{dx(t)}{dt}=A(t)x(t)+B,$$

where

(6) $$\begin{array}{l} A(t)=\left[\begin{array}{ccc} -r_{1}-k(t) & 0 & r_{2}\\ k(t) & -r_{3}-r_{4} & 0\\ 0 & r_{4} & -r_{2} \end{array}\right]\;,\\ \;B=\left[\begin{array}{c} r_{1}M\\ 0\\ 0 \end{array}\right]\text{and}\;x(t)=\left[\begin{array}{c} x(t)\\ y(t)\\ z(t) \end{array}\right]. \end{array}$$

**Table 3:** **Parameters for neurotransmitter release with values identical to those used in publicly available Matlab source code MAP__BS at http://www.essexpsychology.macmate.me/HearingLab/modelling.html**.

**Parameter**	**Description**	**Value**
*r*_1_ (*s*^−1^)	Manufacturing rate	2
*r*_2_ (*s*^−1^)	Restoration rate	100
*r*_3_ (*s*^−1^)	Loss rate	30
*r*_4_ (*s*^−1^)	Recycling rate	150

#### 2.1.3. Stochastic synapse model

Subsequently, Sumner et al. (2002) and Meddis (2006) modified Meddis (1986) to build a model where movement of neurotransmitter is stochastic rather than deterministic and neurotransmitter in the immediate store is quantal rather than continuous. The stochastic Meddis (2006) synapse model is of the following form,

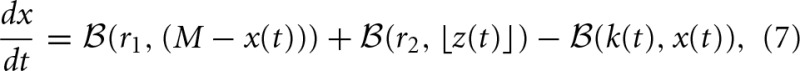

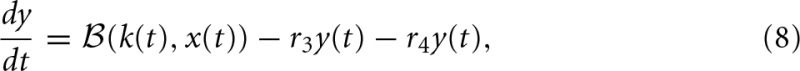

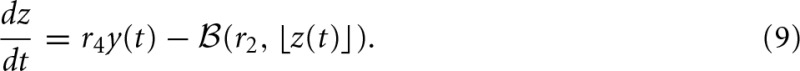







Stochastic movement of discrete vesicles of neurotransmitter is described by the binomial random variable, 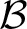
(ρ, *n*): if there are *n* vesicles available during a small *dt*, each with equal probability of moving ρ dt, then 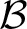
(ρ, *n*) is the number of vesicles moving during *dt*. Vesicles in the immediate store are quantal so *z*(*t*) is mapped to the largest previous integer, ⌊*z*(*t*)⌋.

#### 2.1.4. Phenomenological synapse model

It has been shown that by using rate estimates from a fractional Gaussian noise driven Poisson process model, the shape of published histograms of spontaneous discharge rate (Liberman, 1978) can be reproduced (Jackson and Carney, 2005). This has been incorporated into a phenomenological model of the synapse in the IHC-AN complex by Zilany et al. (2009); Zilany and Carney (2010); Zilany et al. (2014). This synapse model has both exponential and a power-law adaptation functions. The exponential adaptation is implemented using the diffusion model of Westerman and Smith (1988). The exponential adaptation path is followed by two parallel fast and slow power-law adaptation function. The fractional Gaussian noise is incorporated in the slow power-law adaptation path. The input to the synapse model is the relative membrane potential of the inner hair cell.

#### 2.1.5. Models for converting vesicle release to AN spikes

In the deterministic rate model of Meddis (1986), the amount of neurotransmitter in the cleft causes a post-synaptic spike at time *t* with probability,

(10) $$p_{\text{conv}}(t)=hy(t)dt,$$

where *h* is a constant. An absolute refractory period of 1 ms during which no spike can occur is applied. A relative refractory period is not considered.

In the quantal stochastic model of Meddis (2006), each ejected vesicle to the cleft can generate a spike in the auditory nerve after an absolute refractory period (ARP) and relative refractory period (RRP) are considered. If a vesicle is released, a spike in the post-synaptic AN is generated if *p*_conv_(*t*) is greater than a uniformly distributed random number between 0 and 1.

(11) $$p_{\text{conv}}(t)=\{\begin{array}{ll} 0 & \text{if }t-t_{l}<t_{A},\\ 1-C_{r}e^{-(\frac{t\;-\;t_{l}\;-\;t_{A}}{t_{R}})} & \text{if }t-t_{l}\geq t_{A}, \end{array},$$

where *C*_*r*_ = 1, *t*_*R*_ = 0.6 ms is the time constant of relative refractoriness, *t*_*A*_ = 0.75 ms is the ARP, *t* is the current time, and *t*_*l*_ is the time of the previous spike.

The conversion model of Sumner et al. (2002) is very similar to the conversion model in the Meddis (2006) model. The differences are that in the Sumner et al. (2002), *C*_*r*_ = 0.55 and *t*_*R*_ = 0.8 ms.

In the Zilany et al. (2009), Zilany and Carney (2010) and Zilany et al. (2014) spike generator model, spike times in the auditory nerve are generated by a renewal process that simulate a non-homogeneous Poisson process driven by the output of the synapse model.

### 2.2. Previous statistical analysis

#### 2.2.1. Empirical vesicle release distribution

Heil et al. (2007) has shown that the empirical ISI distribution for spontaneous neural activity in cat auditory nerve fibers is better described if the IEI distribution for vesicle release events is a mixture of an exponential distribution and a gamma distribution. The gamma distribution has a shape parameter equal to two, and both the gamma distribution and the exponential distribution have the same scale parameter.

To calculate the ISI parameters, ARP and RRP in the form of Equation (11) are used. Two additional parameters are involved:

- θ is the scale factor for both the exponential distribution and the gamma distribution;
- ρ is the fraction of gamma distribution in the mixture.

Heil et al. (2007) obtained the following equation describing the ISI probability density function (PDF),

(12) $$D(t)=\{\begin{array}{l} \frac{\theta }{t_{R}(\theta -\frac{1}{t_{R}})}((e^{-(\frac{t\;-\;t_{A}}{t_{R}})}-e^{-\theta (t\;-\;t_{A})})(1-\rho +\frac{\rho \theta }{\theta -\frac{1}{t_{R}}})\\ -\rho \theta (t-t_{A})e^{-\theta (t\;-\;t_{A})})\text{ for }t\geq t_{A},\\ 0\text{ for }t<t_{A}, \end{array}$$

#### 2.2.2. Empirical firing correlations

The Fano-factor time curve is a measure of correlation over time. Fano-factor is dispersion in a variable, as a function of an increasing time-window for obtaining data on which to estimate the dispersion. For a spike train, the Fano-factor is the variance of the number of spikes in a time window divided by the mean of number of spikes from a single spike train in that time window. We denote:

- *T* as the size of a specific counting time window.
- *F*(*T*) as the Fano-factor for window size *T*.

**Figure 1:**
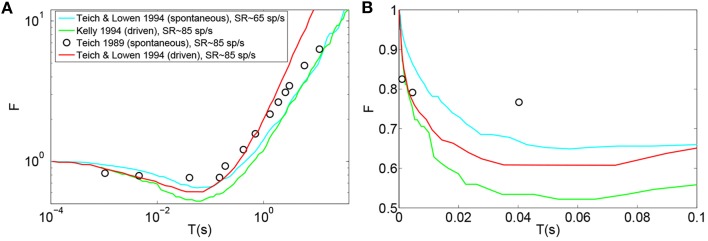
**(A)** Time-window dependent Fano-factor for driven and spontaneous activities in the auditory nerve fiber. Figure created from data in Teich and Lowen (1994), Kelly (1994), Teich (1989). **(B)** Time-window dependent Fano factor of **(A)** on linear axes for time windows shorter than 0.1 s.

Teich and Lowen (1994), Kelly (1994), Teich (1989), Lowen and Teich (1992) plotted empirical Fano-factor time curves for neural activity in mammalian auditory nerve fibers as seen in Figures 1A,B. The Fano-factor is 1 for sufficiently small time windows. It slightly decreases to below 1 over time scales on the order of tens of ms after which it increases monotonically and reaches more than 10 for time windows of a few tens of seconds. It has been shown that negative short term correlation observed in the Fano factor curve of spontaneous activity of a simulated AN fiber model with second order refractory behavior matches the data of Lowen and Teich (1992) for time windows between 15 ms and 100 ms (Gaumond, 2002).

### 2.3. New models

#### 2.3.1. Short-term depression in vesicle release probability (STD_v_)

In AN spontaneous spike trains, the shortest ISIs occur much less frequently than the most likely ISIs (Heil et al., 2007). In Meddis (2006), this feature of ISI statistics is accounted for by ARP and RRP. Given this model includes variable relative refractory times in AN fibers, during which pre-synaptic vesicle release is unaffected, this would mean many vesicles are released that do not give rise to spikes. We therefore seek an alternative model in which what has been attributed to refractoriness is actually mainly due to pre-synaptic effects, due to vesicles not being released at all for durations longer than the absolute refractory period of the ANs. We return to this in Discussion.

Our hypothesis is that all vesicle releases, apart from any that occur during the absolute refractory period, cause action potentials, but that vesicle release is subject to short term depression. We introduce short term depression to pre-synaptic release probability in a manner analogous to standard stochastic models of cortical short term depression (Tsodyks and Markram, 1997; Wang, 1999; Hennig, 2013; McDonnell et al., 2013). In this model, immediately following release, the probability of release drops dramatically and then, increases back to a baseline level over a time frame that matches the spike data.

There are two additional parameters introduced in this model:

- τ_*s*_ is the time constant of short term depression.
- *a* is a fraction indicating an instantaneous decrease in release probability.

The model for the change of *k*(*t*) overtime is

(13) $$\frac{dk(t)}{dt}=\frac{\text{max}(0,bc^{3}(t))-k(t)}{\tau_{s}}+ak(t)\sum_{i}\delta (t-t_{v_{i}}),$$

where *t*_*v*_*i*__ is the time of *i*th release.

#### 2.3.2. Channel noise in inner hair cell calcium channels

Auditory nerve spike trains show positive long term correlation and usually negative short term correlation (Teich, 1989; Lowen and Teich, 1992; Kelly, 1994; Teich and Lowen, 1994). We hypothesize that a possible origin of the correlation is stochastic variability in the inner hair cell calcium channels. A biophysical model and a phenomenological model of calcium channel noise in the inner hair cell are built.

***2.3.2.1. Biophysical model***. In the Meddis (2006) model, long term correlation observed in AN fibers can be partially explained by depletion of readily available vesicles, as explained in the Results section. In the Results section, we show that the Meddis (2006) depleted model with readily available vesicles depleted by decreasing the maximum number of vesicles in the immediate store, or by increasing the spontaneous rate, both require much higher vesicle release rate than the non-depleted Meddis (2006) model.

Other possible origins of the observed long term correlation have been suggested, including fractal ion channel gating (Teich, 1989; Liebovitch and Toth, 1990), fractal behavior of the specialized proteins with direct role in exocytosis (Lowen et al., 1997), self-organized criticality in ion channel gating for example due to ion-conformational interaction (Kharkyanen et al., 1993; Brazhe and Maksimov, 2006), and fractal dynamics of transmitter diffusion in the synaptic junction (Teich, 1989).

An integrate and fire model with renewal point process input has been suggested to be capable of producing long term correlation that matches empirical data from spike trains of cortical neurons (Jackson, 2004). Unlike cortical neurons, inner hair cells encode graded input with a graded membrane potential (Van Steveninck and Laughlin, 1996). We aim to cast light on the possible biophysical mechanisms in the IHC-AN complex that can produce renewal point processes and hence long term correlation in the spike trains of auditory nerves.

Meddis (2006) assumes the calcium concentration dependence of the release probability to be due to voltage dependent calcium channels. We hypothesize that a possible biophysical mechanism of the fractional Gaussian noise in Jackson and Carney (2005); Zilany et al. (2009); Zilany and Carney (2010); Zilany et al. (2014) is the random fluctuations in the number of open and closed calcium ion channels as they are expected to cause variability in vesicle release probabilities.

We introduce to the Meddis (2006) model a four-state model of channel gating with standard transition rate formulae (Goldwyn and Shea-Brown, 2011; Schmerl and McDonnell, 2013),

(14) $$\alpha (v)=\frac{1}{\tau_{m}\left(1+\frac{e^{-\gamma v(t)}}{\beta }\right)},$$

(15) $$\beta (v)=\frac{1}{\tau_{m}}-\alpha (v).$$

Equation (2) therefore changes to

(16) $$\frac{dc(t)}{dt}=-\frac{c(t)}{\tau_{c}}+\nu g_{c}n(t)(E_{c}-v(t)),$$

where *n*(*t*) is the number of open calcium channels out of total of *N* calcium channels and *g*_*c*_ is the single calcium channel conductance.

***2.3.2.2. Phenomenological model***. We consider a phenomenological model of calcium channel noise that we add to the Meddis (2006) model. Instead of modeling discrete channel noise, we add an Ornstein Uhlenbeck process to the mean fraction of open calcium channels, *m*^3^(*t*). Equation (2) changes to:

(17) $$\frac{dc(t)}{dt}=-\frac{c(t)}{\tau_{c}}+\nu G_{c}(f(m^{3}(t)+X(t)))(E_{c}-v(t)),$$

where *f*(·) := max(0, min(1, ·)) ensures the fraction of open channels is restricted to the interval [0, 1] and *X*(*t*) is a noise driven from Ornstein Uhlenbeck process; i.e.,

(18) $$dX(t)=\frac{dt}{\tau_{o}}(\mu_{o}-X(t))+\sigma_{o}dW_{t},$$

where *W*_*t*_ denotes the Wiener process and the mean (μ_*o*_), time constant (τ_*o*_) and variance (σ_*o*_) of the noise are positive constants.

#### 2.3.3. Noise in inner hair cell membrane potential

We also consider an alternative phenomenological model of noise where the IHC membrane potential is subject to an additive Ornstein Uhlenbeck process. Equation (2) changes to:

(19) $$\frac{dc(t)}{dt}=-\frac{c(t)}{\tau_{c}}+\nu G_{c}m^{3}(t)(E_{c}-v(t)-X(t)),$$

#### 2.3.4. Combination of short term depression in vesicle release model and phenomenological calcium channel noise model

A possible origin of short term correlation in AN spike trains is a form of refractoriness (Teich and Lowen, 1994). We introduce a model that combines short term depression in vesicle release and phenomenological calcium channel noise as follows

(20) $$\frac{dk(t)}{dt}=\frac{k_{1}(t)-k(t)}{\tau_{s}}+ak(t)\sum_{i}\delta (t-t_{v_{i}}),$$

where *k*_1_(*t*) is the vesicle release rate when Ornstein Uhlenbeck noise is added as calcium channel noise in the Meddis (2006) model.

### 2.4. Parameters

The parameters in Table 1 (except *g*_*c*_), in Table 3, and for *t*_*A*_ and *t*_*R*_ (except in Table 5) were obtained from publicly available Matlab source code MAP__BS at http://www.essexpsychology.macmate.me/HearingLab/modelling.html. The parameters in Table 2 and for *g*_*c*_ were obtained from the literature (Hudspeth and Lewis, 1988; Zampini et al., 2013). The values of *b* and *bc*(*t*)^3^ were chosen in order to produce the desired spontaneous rates in AN fibers. In Table 5, the parameters τ_*s*_, *t*_*R*_ and *a* were obtained through parameter searches, in order to obtain a close quantitative fit to the data of Heil et al. (2007), while keeping estimated values of θ and ρ close to the results of Heil et al. (2007). The parameters τ_*o*_, σ_*o*_ and μ_*o*_ were chosen to produce spontaneous activity in the auditory nerve that qualitatively matches the empirical Fano factor data of Teich and Lowen (1994).

**Table 4:** **Values for depletion of available vesicles as a possible source of long term correlation in the original Meddis (2006) model**.

*****M*****	**SR (spikes.s^−1^)**	**Trace**	***k* (s^−1^)**	**Short term correlation**	**Long term correlation**
20	~65	Blue	5	Slight	No
6	~65	Red	107	Yes	Partial
20	~160	Green	55	Yes	Partial

**Table 5:** **Comparison of the original Meddis (2006) model and Meddis (2006) model with relative refractoriness in the auditoy nerve substituted by short term depression in vesicle release**.

**Model**	**Trace**	***t*_*A*_ (ms)**	***t*_*R*_ (ms)**	**τ_*s*_ (ms)**	**b*c*^3^ (s^−1^)**	**a**	**θ (ms^−1^)**	**ρ**	**Log likelihood**
Original Meddis	Orange	0	0	NA	NA	NA	0.04	0	−1.11× 10^6^
Original Meddis	Green	0	3.5	NA	NA	NA	0.05	0.44	−1.08× 10^6^
Meddis with STD_v_	Black	0	NA	3	6	0.001	0.05	0.37	−1.08× 10^6^
Meddis with STD_v_	Blue	0.75	NA	2.5	5	0.001	0.05	0.37	−1.08× 10^6^

*M = 20 and SR~65 spikes per second. For fitting to Equation (12), in the Equation (12) *t*_*A*_ = 0.75 ms and *t*_*R*_ = 3.5 ms were used. Log likelihood was used as a measure of goodness of fit*.

The maximum number of readily releasable vesicles in the immediate store, *M*, in the Meddis (2006) model is considered to be 10. Moser and Beutner (2000) suggested the average number of vesicles in the immediate store to be about 14 vesicles per active zone. (Khimich et al., 2005) suggested a readily release pool of about 22 docked vesicles in the IHC of mouse. Pangršič et al. (2010) estimated a readily releasable pool of 12 vesicles per active zone in the pachanga mouse. We assumed the maximum readily available pool size, *M*, to be 20 vesicles per active zone.

## 3. Results

### 3.1. Previous models

**Figure 2:**
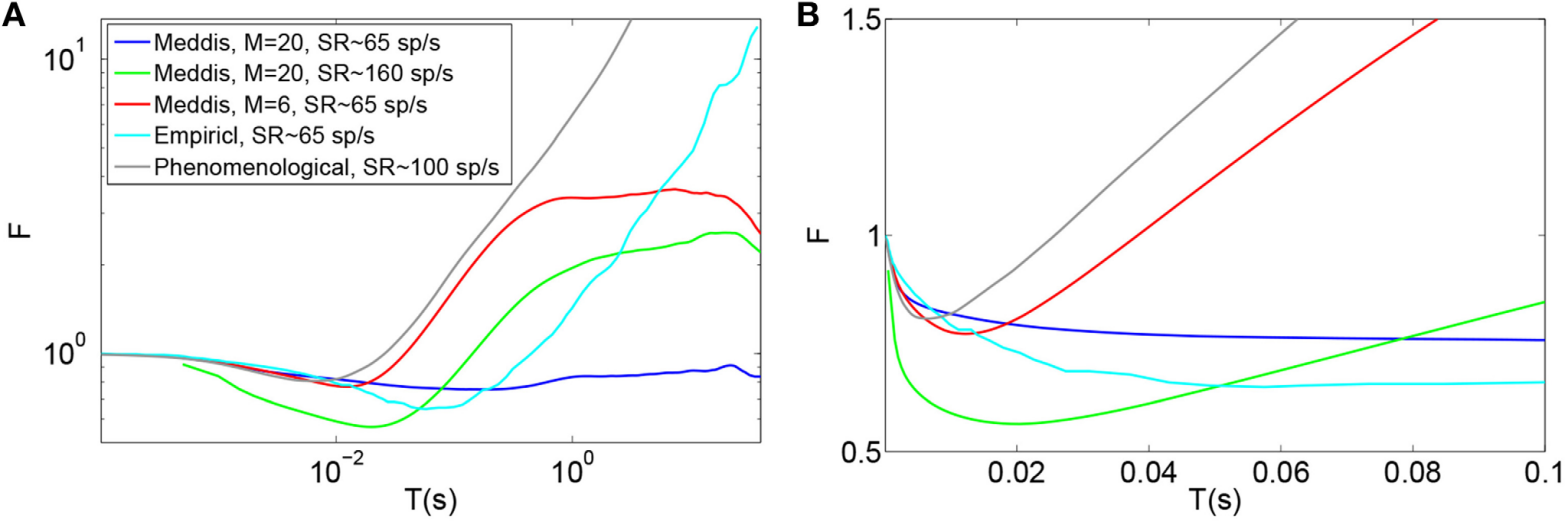
**Time window dependent Fano factor for spontaneous activity in the auditory nerve obtained from previous models. (A)** Gray: Fano factor time curve for a spike train generated by the Zilany et al. (2014) model with SR~100 spikes per second. Blue, green, red: in the original Meddis (2006) model, by decreasing the maximum number of available vesicles from M = 20 with SR~65 spikes per second (Blue) to M = 6 with SR~65 spikes per second (Red) or increasing the spontaneous firing rate from SR~65 spikes per second with M = 20 to SR~160 spikes per second with M = 20 (Green), short term negative correlation starts at smaller time windows and long term positive correlation is increased. Light blue: Empirical data from spontaneous activity in auditory nerve with SR~ 65 spikes per second created from data in Teich and Lowen (1994). **(B)** Time-window dependent Fano factor of **(A)** on linear axes for time windows shorter than 0.1 s. Parameters are summarized in Table 4.

Figures 2A (Gray) and **2B** (Gray) show the Fano factor time curve of a spike train generated by the (Zilany et al., 2014) synapse model. These figures are obtained by running the model code available at http://www.urmc.rochester.edu/labs/Carney-Lab/publications/auditory-models.cfm, with a relative membrane voltage input of 0 V. Like the empirical Fano factor of Figure 2A(Light blue), the Fano factor increases to about 10 for large counting time windows. The Fano factor in Figure 2A (Gray) does not decrease below one for shorter time windows as much as the empirical Fano factor shown in Figure 2A (Light blue) does. The time scales of the correlation do not match empirical data of Figure 2A (Light blue).

In the Meddis (2006) model, long term correlation observed in the auditory nerves can be partially explained by depletion of readily available vesicles in the immediate store. In Figures 2A,B, the blue trace is the Fano factor time curve for the original Meddis (2006) model with a maximum number of readily available vesicles of *M* = 20 and a spontaneous rate of around 65 spikes per second. The red trace is the Fano factor time curve for the original Meddis (2006) model with a maximum number of readily available vesicles of *M* = 6 and a spontaneous rate of around 65 spikes per second. The green trace is the Fano factor time curve for the original Meddis (2006) model with a maximum number of readily available vesicles of *M* = 20 and a spontaneous rate of about 160 spikes per second. The Fano factor curves in red and green increase to higher values than the Fano factor curve in blue does for large time windows and are a better qualitative match to the empirical data of Figure 2A (Light blue). The Fano factor does not reach 10 for sufficiently large time windows. The magnitude of the decrease in Fano factor below one for shorter time windows is comparable to the empirical data of Figure 2A (Light blue.)

As shown in Figure 2A, long term correlation in the (Meddis, 2006) model can be partially produced if either the maximum number of releasable vesicles is decreased or the firing rate is increased, both of which cause depletion of available vesicles in the immediate store. In this model, low spontaneous rate fibers are associated with smaller pools of vesicles, and high spontaneous rate fibers are associated with larger pools of vesicles. In the depleted model, the time scales of the correlation do not match the empirical data of Figure 2A (Light blue). Depletion of vesicles moves the onset of short and long term correlations to smaller time windows.

Depletion of available vesicles in the (Meddis, 2006) model (by decreasing the maximum number of available vesicles from 20 with SR of about 65 spikes per second to 6 with SR of about 65 spikes per second, or by increasing the spontaneous rate to around 160 spikes per second with maximum number of available vesicles of 20) produces an average vesicle release rate, *k*, of 107 and 55 (s^−1^) respectively that are both much larger than 5 (s^−1^), which is the *k* of the Meddis (2006) model with a maximum number of available vesicles of 20 and the spontaneous rate of about 65 spikes per second.

### 3.2. Short-term depression model

Here we consider the case where the relative refractoriness component of the Meddis (2006) model is removed and we use our alternative model of short term depression in vesicle release probability. That is, the release rate in the Meddis (2006) model, *k*(*t*), as given by Equation (1), was replaced by *k*(*t*) of Equation (13), and relative refractoriness in the auditory nerve was omitted. Using this model, ISI data for spontaneous activity in an AN fiber was simulated.

**Figure 3:**
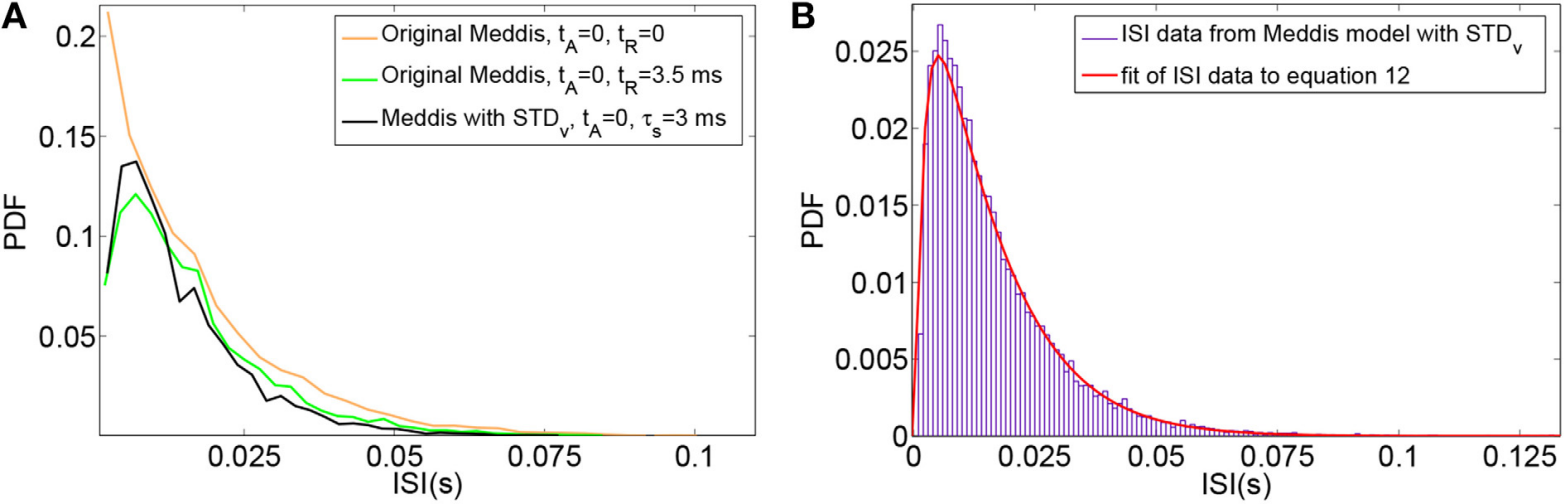
**(A)** PDF of ISIs for the original Meddis (2006) model with *t*_*A*_ = 0 and *t*_*R*_ = 0 (Orange), for the original Meddis (2006) model with *t*_*A*_ = 0 and *t*_*R*_ = 3.5 ms (Green) and for Meddis (2006) model with relative refractoriness in the auditory nerve substituted by short term depression in vesicle release where *t*_*A*_ = 0, τ_*s*_ = 3 ms and *bc*^3^ = 6 s^−1^ (Black). **(B)** Fitting PDF of ISI data for Meddis (2006) model with relative refractoriness in the auditory nerve substituted by short term depression in vesicle release where *t*_*A*_ = 0.75 ms, τ_*s*_ = 2.5 ms and *bc*^3^ = 5 s^−1^ to Equation (12). In all traces, SR~65 spikes per second. Parameters are summarized in Table 5.

The effect of substituting relative refractoriness in the auditory nerve with short term depression in vesicle release in the Meddis (2006) model is more clearly observed in the simulated data when the absolute refractory period is (unrealistically) assumed to be zero. Under this assumption, Figure 3A shows that in the Meddis (2006) model, similar to including relative refractoriness in the auditory nerve, the alternative model of short term depression in vesicle release leads to the least probable ISIs being larger than they would otherwise be.

A distribution fitting application which returns maximum likelihood estimations of the model parameters was used to estimate the parameters that produce the best fit of the simulated ISIs to the empirical results. Figure 3B shows that the PDF of the simulated data for the Meddis (2006) model with AN relative refractoriness replaced by short term depression in vesicle release in blue and the best fit to Equation (12) in red. The refractory time constants, *t*_*A*_ and *t*_*R*_, were kept at fixed values. The free parameters, θ and ρ, were estimated.

The models in Figures 3A,B were fitted to Equation (12), and the corresponding values of θ and ρ were estimated and summarized in Table 5. Parameters τ_*s*_ and *a* were obtained through parameter search in order to obtain a good fit to data while keeping θ and ρ close to the result of Heil et al. (2007).

In two different neurons, Heil et al. (2007) obtained θ = 0.0988 (ms^−1^) and ρ = 0.39 for *t*_*A*_ = 0.69 ms, and *t*_*R*_ = 0.58 ms when SR = 65 spikes per second and θ = 0.0862 (ms^−1^) and ρ = 0.43 for *t*_*A*_ = 0.73 ms, and *t*_*R*_ = 0.41 ms when SR = 57.1 spikes per second. Using the short term depression in vesicle release model, we estimated θ and ρ to be 0.05 (ms^−1^) and 0.37, respectively. Thus, Heil et al. (2007) scaling factors, θ, and fraction of gamma distribution in the mix, ρ, are comparable to what we obtained with our model with comparable spontaneous rate.

However, while (Heil et al., 2007) assumed the post-synaptic refractory period to be less than 1 ms, we obtain our result with a post-synaptic refractory period of a few milliseconds. Despite this difference, our model has introduced three features to the model's ISI distribution that are common with the data of Heil et al. (2007): an ISI PDF with a single maxima such that the PDF increases from zero to its peaks for small ISIs just above the absolute refractory period, a comparable scale factor and a comparable fraction of gamma distribution in the mix of exponential and gamma distributions.

### 3.3. Calcium channel noise

#### 3.3.1. Biophysical model

**Figure 4:**
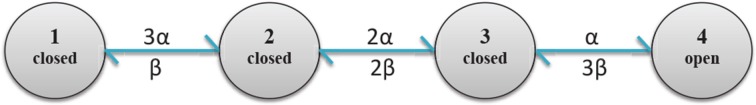
**Diagram of calcium channel states and transition rates**. States 1, 2, 3 and 4, respectively have 0, 1, 2 and 3 open subunits. State 4 is the only conducting state.

Here we consider the case where the biophysical model of calcium channel noise is added to the Meddis (2006) model. That is, in the Meddis (2006) model the state of each of *N* calcium channels is simulated, stochastic changes of states based on the state diagram of Figure 4 are permitted, and *c*(*t*) given in Equation (2) is replaced by *c*(*t*) given by Equation (16).

**Figure 5:**
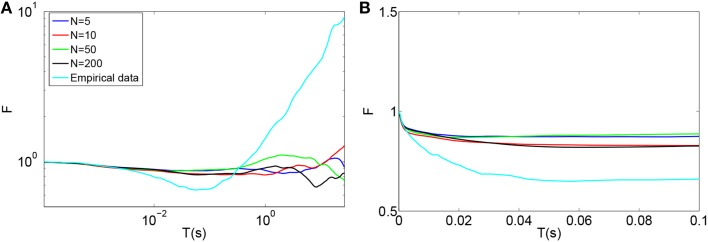
**(A)** Time-window dependent Fano factor for spontaneous activity in an auditory nerve fiber model using the biophysical model of calcium channel noise in the IHC-AN complex applied to the Meddis (2006) model for different numbers of calcium channels, *N*. Light blue: Empirical data from spontaneous activity in auditory nerve with SR~65 spikes per second created from data in (Teich and Lowen, 1994). **(B)** Time-window dependent Fano factor of **(A)** on linear axes for time windows shorter than 0.1 s. Parameters are summarized in Table 6.

Using this model, the time-window dependent Fano factor of spike counts in the auditory nerve model for different numbers of calcium channels were obtained and shown in Figures 5A,B. Unlike the empirical data of Figure 5A (Light blue), the Fano factor does not increase steadily to a value around 10 for long time windows. A slight decrease in Fano factor for shorter time windows is observed.

In the hair cells of a chick's cochlea, for each hair cell, around 100 calcium channels for short hair cells and 341 for tall hair cells are suggested (Martinez-Dunst et al., 1997), which in turn suggest quite small numbers of channels per synapse. In our model, no improvement was seen in long term correlation by decreasing the number of calcium channels from 200 (Black) to 50 (Green), 10 (Red) and 5 (Blue). We conclude that this calcium channel model fails to add a long term correlation to the spike trains of the auditory nerve in the Meddis (2006) model in a way that matches experimental observations shown in Figure 5A (Light blue).

Adding the biophysical calcium channel model with parameters summarized in Table 6 to the Meddis (2006) model produces *k* of 4, 6, 5 and 4 (s^−1^) respectively for 5, 10, 50, and 200 calcium channels which are all close to 5 (s^−1^), the *k* of the original (Meddis, 2006) model with a spontaneous rate around 65 spikes per second and a maximum number of available vesicles of *M* = 20.

**Table 6:** **Parameters of Meddis (2006) model with biophysical calcium channel noise**.

***N***	**Trace**	***k* (s^−1^)**	**Short term correlation**	**Long term correlation**
5	Blue	4	Slight	No
10	Red	6	Slight	No
50	Green	5	Slight	No
200	Black	4	Slight	No

*M = 20 and SR~65 spikes per second*.

#### 3.3.2. Phenomenological model

Here we consider the case where the phenomenological model of calcium channel noise is added to the Meddis (2006) model. That is, in the Meddis (2006) model, Equation (2) is replaced by Equation (17).

**Figure 6:**
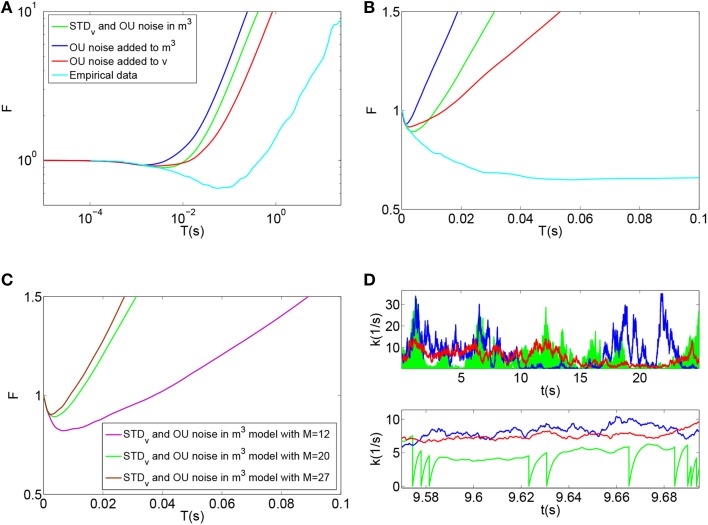
**(A)** Time-window dependent Fano factor for spontaneous activity in an auditory nerve model using Red: phenomenological model of membrane potential noise in IHC-AN complex applied to the Meddis (2006) model. Blue: Phenomenological calcium channel noise model applied to the Meddis (2006) model. Green: adding a combination model of the phenomenological channel noise and short term depression in vesicle release to Meddis (2006) model. Light blue: Empirical data from spontaneous activity in an auditory nerve fiber with SR~65 spikes per second created from data in Teich and Lowen (1994). **(B)** Time-window dependent Fano-factor of **(A)** on linear axes for time windows shorter than 0.1 s. **(C)** Fano factor for the Meddis (2006) model with the combination of phenomenological channel noise and short term depression in vesicle release for different maximum numbers of vesicles in the immediate store on linear axes for time windows shorter than 0.1 s. **(D)** Vesicle release rate for **(A)**. The color representations are the same as in **(A)**. The two subfigures in **(D)** are for different time scales, i.e., the bottom subfigure is a zoom into the top subfigure. The rapid decreases in the green trace in the **(D)** bottom plot for the combination model of phenomenological channel noise and short term depression in vesicle release are due to actual vesicle release while remaining fluctuations are due to channel noise. Parameter are summarized in Tables 7, 8.

Using this model, the time-window dependent Fano factor of spike counts in the auditory nerve model were obtained and shown in Figures 6A,B (Blue). It can be seen in Figure 6A (Blue) that, like empirical Fano factor of Figure 6A (Light blue), the Fano factor increases to about 10 for large counting time windows. But, the Fano factor in Figure 6A (Blue) does not decrease below one for shorter time windows as much as the empirical Fano factor shown in Figure 6A (Light blue) does.

Adding the phenomenological channel noise with parameters summarized in Table 7 to the Meddis (2006) model produces *k* of 7 (s^−1^) which is close to 5 (s^−1^), the *k* of the original Meddis (2006) model with a spontaneous rate around 65 spikes per second and maximum number of available vesicles of *M* = 20.

**Table 7:** **Parameters for the phenomenological models of stochastic variability in the IHC-AN complex**.

**Meddis model with**	**trace**	**τ_*o*_ (s)**	**σ_*o*_**	**μ_*o*_**	***k* (s^−1^)**	**τ_*s*_ (ms)**	***b*c^3^ (ms)**	***a***	**Short term correlation**	**Long term correlation**
OU noise added to *m*^3^	Blue	1.2	0.3	0.38	7	NA	NA	NA	Slight	Yes
OU noise added to v	Red	2	0.04	0	5	NA	NA	NA	Slight	Yes
STD_v_ and OU in *m*^3^	Green	1.2	0.3	0.38	5	2.5	8.5	0.001	Slight	Yes

**M* = 20, and SR~65 spikes per second*.

### 3.4. Combining short-term depression and calcium channel noise

Here we consider a combination of short term depression in vesicle release with the phenomenological model of channel noise within the Meddis (2006) model. That is, in the Meddis (2006) model, *k*(*t*) from Equation (1) was replaced by *k*(*t*) from Equation (20) and relative refractoriness in the auditory nerve in the AN fiber was omitted.

Figures 6A,B (Green) show the time-window dependent Fano factor for auditory nerve fiber spike counts for this model. The Fano factor for this model increases steadily to about 10 for large counting time windows. It can be seen in Figure 6B (Green) that for counting time windows of a few milliseconds, Fano factor decrease is slightly more than that of Figure 6A (Blue) and hence a better match to the empirical data of Figure 6A (Light blue).

Adding the combination model of phenomenological channel noise and short term depression in vesicle release with parameters summarized in Table 7 to the Meddis (2006) model produces *k* of 5 (s^−1^) which is the same as the *k* of the original Meddis (2006) model with a spontaneous rate around 65 spikes per second and maximum number of available vesicles of *M* = 20.

As the maximum number of available vesicles in the immediate store decreases, as shown in Figure 6C, the corresponding minima in the Fano factor curve for shorter time windows increases and the short and long term correlations compare quantitatively to the results from the Zilany et al. (2014) model. *k* = 10 remains close to the *k* = 5 from the original (Meddis, 2006) model with a spontaneous rate around 65 spikes per second and a maximum number of available vesicles of *M* = 20.

This combination model produces a release rate for which the baseline level is mainly controlled by Ornstein Uhlenbeck noise and the post release behavior is mainly controlled by short term depression in vesicle release as shown in Figure 6D (Green).

### 3.5. Comparison of calcium channel noise with membrane potential noise

Here we consider the inclusion of the phenomenological model of noise in the inner hair cell membrane potential model in (Meddis, 2006) model. That is, in the Meddis (2006) model, Equation (2) is replaced by Equation (19).

The time-window dependent Fano factor for AN spike counts in this model is shown in Figures 6A,B (Red). Like the situation of Figure 6A (Blue) where the Ornstein-Uhlenbeck noise is instead included as calcium channel noise, the Fano factor increases steadily to 10 for larger counting time windows, but it decreases below unity less than the empirical Fano factor of Figure 6A (Light blue) for smaller counting time windows.

Adding the phenomenological membrane potential noise with parameters summarized in Table 7 to the Meddis (2006) model produces *k* of 5 (s^−1^) which is the same as the *k* of the original Meddis (2006) model with a spontaneous rate around 65 spikes per second and a maximum number of available vesicles of *M* = 20.

## 4. Discussion

We have shown that adding a combination of short term depression in vesicle release, and time-correlated channel noise, to the existing model of Meddis (2006) results in qualitatively similar results for spontaneous inter-spike interval correlations observed in empirical data. We make the case that it is the qualitative features of the Fano factor curve (namely the occurrence of positive and negative correlations, and the order of magnitude of the positive correlation) that are of most interest. Our model generates auditory nerve spontaneous spike trains for which the spike-count Fano-factor matches empirical data at short and long time scales qualitatively. The qualitative features of the Fano factor curve obtained from the proposed models are summarized in the last columns of Tables 4, 6–8. However, the time scales of maximum negative correlation, and the onset of positive correlation do not exactly match the data. Moreover, the long term correlation in the biophysical model of IHC calcium channel noise does not match empirical data. There are several reasons for these discrepancies. First, the simulation data is only as good as the overall model, which omits many details of the complex calcium channel dynamics of ribbon synapses. We have seen, for example, that a standard biophysical model of channel noise does not induce long-term correlations, while replacing that model with a phenomenological model based on Ornstein-Uhlenbeck noise does so. We suggest that a more biophysically detailed model of calcium channel noise can improve the long term correlation to match empirical data. For example, a model where a single calcium channel controls vesicle release at each docking site (Weber et al., 2010) could potentially lead to a more complicated release dynamics and might produce long term correlation in the auditory nerve spontaneous spiking activity. A second reason might be that the parameters we used (including parameters in the Meddis (2006) model) need to be better tuned to match the empirical data. We left this for future work.

**Table 8:** **Parameters of the combination model of phenomenological channel noise and short term depression in vesicle release probability with various maximum numbers of vesicles in the available store**.

*****M*****	**Trace**	***bc*^3^ (s^−1^)**	***k* (s^−1^)**	**Short term correlation**
12	Purple	5	10	More than *M* = 20
20	Green	8.5	5	Slight
27	Brown	8.5	3	Less than *M* = 20

*τ_*o*_ = *1.2* ms, σ_*o*_ = *0.3*, μ_*o*_ = *0.38*, τ_*s*_ = *2.5* ms, *a* = *0.001* and SR~65 spikes per second*.

There are several justifications for replacing auditory nerve relative refractoriness with short term depression in vesicle release probability in the model. First, extensive neurotransmitter release can be toxic to neural tissues and cleaning up the excessive transmitters by glia cells requires a large amount of energy (Glowatzki et al., 2006). Short term depression in vesicle release will reduce the number of vesicles released, which in turn will reduce potential for toxicity and energy usage. Moreover, since it is thought that single vesicle produces spikes in AN fibers, for energetics reasons it is wasteful to release vesicles during the refractory period when spikes cannot occur.

A possible mechanism for short term depression in vesicle release could be the presence of auto-inhibitory metabotropic receptors called auto-receptors (Billups et al., 2005). To our knowledge, however, there is no evidence either for or against the presence of such auto-receptors in inner hair cells. Alternatively, it is possible that complex intra-cellular calcium dynamics and its relationship to vesicle exocytosis could cause such effects.

We hypothesize that observations of variable minimum time between spikes attributed to “relative refractoriness” above) in the IHC-AN complex is mainly due to pre-synaptic effects, namely that vesicle release sometimes doe not occur for a period longer than are the absolute refractory period. However, it is also possible that actual relative refractoriness in auditory nerve recovery following a spike (Cartee et al., 2000), and short term depression in vesicle release probability in the ribbon synapse could co-exist.

To obtain a fit close to the data of Heil et al. (2007), we have chosen the time constant of short term depression in the vesicle release to be 2.5 ms. Short term depression in vesicle release has been observed in synapses other than the ribbon synapse of inner hair cells (e.g., Stevens and Wang, 1995; Hjelmstad et al., 1997). Whole cell recordings from hippocampal pyramidal neurons showed that a 20 ms refractory period was required between vesicle releases (Stevens and Wang, 1995). In a different experiment, Hjelmstad et al. (1997) observed a 6–7 ms period following release during which the synapse was incapable of transmission. Consequently, the time-scale of 2.5 ms is potentially biologically plausible.

In this paper we aimed to simulate auditory nerve spontaneous spiking patterns that provided an improved statistical match to empirical data. We modified a revised version of the Meddis (2006) model to develop a more biophysically detailed description of stochastic variability in the IHC-AN complex. It has been suggested (Morse and Evans, 1996; McDonnell et al., 2008) that significantly decreased stochastic variability in AN spiking generated by cochlear implants is a contributing factor to imperfect performance of these implants. A potential application of our model, therefore, is as a component in a larger model of the auditory system designed to predict differences in neural activity in higher brain regions, such as the cochlear nucleus, due to electrical stimulation by cochlear implants, in comparison with natural acoustic stimulation.

Based on our findings it will be interesting for future work to build on our study with a more detailed model of the calcium dynamics of the ribbon synapse in inner hair cells. Such a model might be capable of explaining both pre-synaptic short-term depression in vesicle release, and long-term correlations due to calcium fluctuations.

## Funding

Mark D. McDonnell's contribution was supported by the Australian Research Council under ARC grant DP1093425 (including an Australian Research Fellowship).

### Conflict of interest statement

The authors declare that the research was conducted in the absence of any commercial or financial relationships that could be construed as a potential conflict of interest.

## References

[B1] Billups B. Graham B. P. Wong A. Y. Forsythe I. D. (2005). Unmasking group III metabotropic glutamate auto-receptor function at excitatory synapses in the rat CNS. J. Physiol. 565, 885–896. 10.1113/jphysiol.2005.086736 15845577 PMC1464548

[B2] Brazhe A. R. Maksimov G. V. (2006). Self-organized critical gating of ion channels: on the origin of long-term memory in dwell time series. Chaos Int. J. Nonlin. Sci. 16, 033129. 10.1063/1.2355657 17014234

[B3] Cartee L. A. van den Honert C. Finley C. C. Miller R. L. (2000). Evaluation of a model of the cochlear neural membrane. I. Physiological measurement of membrane characteristics in response to Intra-meatal electrical stimulation. Hear. Res. 146, 143–152. 10.1016/S0378-5955(00)00109-X 10913891

[B4] Gaumond R. P. (2002). Ratio of variance to mean of action potential counts for an auditory nerve fiber model with second order refractory behavior. J. Acoust. Soc. Am. 93, 2035–2037. 10.1121/1.406717 8473615

[B5] Glowatzki E. Fuchs P. A. (2002). Transmitter release at the hair cell ribbon synapse. Nat. Neurosci. 5, 147–154. 10.1038/nn796 11802170

[B6] Glowatzki E. Cheng N. Hiel H. Yi E. Tanaka K. Ellis-Davies E. C. R.. (2006). The glutamate-aspartate transporter glast mediates glutamate uptake at inner hair cell afferent synapses in the mammalian cochlea. J. Neurosci. 26, 7659–7664. 10.1523/JNEUROSCI.1545-06.2006 16855093 PMC6674291

[B7] Goldwyn J. H. Shea-Brown E. (2011). The what and where of adding channel noise to the Hodgkin-Huxley equations. PLoS Comput. Biol. 7:e1002247. 10.1371/journal.pcbi.1002247 22125479 PMC3219615

[B8] Heil P. Neubauer H. Irvine D. R. Brown M. (2007). Spontaneous activity of auditory-nerve fibers: insights into stochastic processes at ribbon synapses. J. Neurosci. 27, 8457–8474. 10.1523/JNEUROSCI.1512-07.2007 17670993 PMC6673073

[B9] Hennig M. H. (2013). Theoretical models of synaptic short term plasticity. Front. Comput. Neurosci. 7:45. 10.3389/fncom.2013.00045 23626536 PMC3630333

[B10] Hjelmstad G. O. Nicoll R. A. Malenka R. C. (1997). Synaptic refractory period provides a measure of probability of release in the hippocampus. Neuron 19, 1309–1318. 10.1016/S0896-6273(00)80421-3 9427253

[B11] Hodgkin A. L. Huxley A. F. (1952). A quantitative description of membrane current and its application to conduction and excitation in nerve. J. Physiol. 117, 500–544. 12991237 10.1113/jphysiol.1952.sp004764 PMC1392413

[B12] Hudspeth A. J. Lewis R. S. (1988). Kinetic analysis of voltage-and ion-dependent conductances in saccular hair cells of the bull-frog, Rana catesbeiana. J. Physiol. 400, 237–274. 2458454 10.1113/jphysiol.1988.sp017119 PMC1191806

[B13] Jackson B. S. Carney L. H. (2005). The spontaneous-rate histogram of the auditory nerve can be explained by only two or three spontaneous rates and long-range dependence. J. Assoc. Res. Otolaryngol. 6, 148–159. 10.1007/s10162-005-5045-6 15952051 PMC2538337

[B14] Jackson B. (2004). Including long-range dependence in integrate-and-fire models of the high interspike-interval variability of cortical neurons. Neural Comput. 16, 2125–2195. 10.1162/0899766041732413 15333210

[B15] Johnson S. L. Franz C. Knipper M. Marcotti W. (2009). Functional maturation of the exocytotic machinery at gerbil hair cell ribbon synapses. J. Physiol. 587, 1715–1726. 10.1113/jphysiol.2009.168542 19237422 PMC2683959

[B16] Kelly O. E. (1994). Analysis of Long-Range Dependence in Auditory-Nerve Fiber Recordings. Master's Thesis, Rice University, Houston.

[B17] Kharkyanen V. N. Panchouk A. S. Weinreb G. E. (1993). Self-organization effects induced by ion-conformational interaction in biomembrane channels. J. Biol. Phys. 19, 259–272. 10.1007/BF007006657535566

[B18] Khimich D. Nouvian R. Pujol R. tom Dieck S. Egner A. Gundelfinger E. D.. (2005). Hair cell synaptic ribbons are essential for synchronous auditory signalling. Nature 434, 889–894. 10.1038/nature03418 15829963

[B19] Liberman M. C. (1978). Auditory nerve response from cats raised in a low noise chamber. J. Acoust. Soc. Am. 63, 442–455. 10.1121/1.381736 670542

[B20] Liebovitch L. S. Toth T. I. (1990). Using fractals to understand the opening and closing of ion channels. Anna. Biomed. Eng. 18, 177–194. 10.1007/BF02368428 1693478

[B21] Lowen S. B. Teich M. C. (1992). Auditory nerve action potentials form a nonrenewal point process over short as well as long time scales. J. Acoust. Soc. Am. 92, 803–806. 10.1121/1.403950 1324263

[B22] Lowen S. B. Cash S. S. Poo M. M. Teich M. C. (1997). Quantal neurotransmitter secretion rate exhibits fractal behavior. J. Neurosci. 17, 5666–5677. 9221766 10.1523/JNEUROSCI.17-15-05666.1997 PMC6573209

[B23] Martinez-Dunst C. Michaels R. L. Fuchs P. A. (1997). Release sites and calcium channels in hair cells of the chicks cochlea. J. Neurosci. 17, 9133–9144. 9364060 10.1523/JNEUROSCI.17-23-09133.1997 PMC6573622

[B24] Matthews G. Fuchs P. (2010). The diverse roles of ribbon synapses in sensory neurotransmission. Nat. Rev. Neurosci. 11, 812–822. 10.1038/nrn2924 21045860 PMC3065184

[B25] McDonnell M. D. Stocks N. G. Pearce C. E. M. Abbott D. (2008). Stochastic Resonance: From Suprathreshold Stochastic Resonance to Stochastic Signal Quantization. Cambridge: Cambridge University Press 10.1017/CBO9780511535239

[B26] McDonnell M. D. Mohan A. Stricker C. (2013). Mathematical analysis and algorithms for efficiently and accurately implementing stochastic simulations of short-term synaptic depression and facilitation. Front. Comput. Neurosci. 7:58. 10.3389/fncom.2013.00058 23675343 PMC3650633

[B27] Meddis R. (1986). Simulation of mechanical to neural transduction in the auditory receptor. J. Acoust. Soc. Am. 79, 709–711. 10.1121/1.393460 2870094

[B28] Meddis R. (2006). Auditory-nerve first-spike latency and auditory absolute threshold: a computer model. J. Acoust. Soc. Am. 119, 406–417. 10.1121/1.2139628 16454295

[B29] Morse R. P. Evans E. F. (1996). Enhancement of vowel coding for cochlear implants by addition of noise. Nat. Med. 2, 928–932. 10.1038/nm0896-928 8705865

[B30] Moser T. Beutner D. (2000). Kinetics of exocytosis and endocytosis at the cochlear inner hair cell afferent synapse of the mouse. Proc. Natl. Acad. Sci. U.S.A. 97, 883–888. 10.1073/pnas.97.2.883 10639174 PMC15425

[B31] Mulroy M. J. Altmann D. W. Weiss T. F. Peake W. T. (1974). Intracellular electric responses to sound in a vertebrate cochlea. Nature 249, 482–485. 10.1038/249482a0 4834237

[B32] Pangršič T. Lasarow L. Reuter K. Takago H. Schwander M. Riedel D.. (2010). Hearing requires otoferlin-dependent efficient replenishment of synaptic vesicles in hair cells. Nat. Neurosci. 13, 869–876. 10.1038/nn.2578 20562868

[B33] Schmerl B. A. McDonnell M. D. (2013). Channel-noise-induced stochastic facilitation in an auditory brainstem neuron model. Phys. Rev. E 88:052722. 10.1103/PhysRevE.88.052722 24329311

[B34] Scott P. C. Cowan A. I. Stricker C. (2012). Quantifying impacts of short-term plasticity on neuronal information transfer. Phys. Rev. E 85:041921. 10.1103/PhysRevE.85.041921 22680512

[B35] Stevens C. F. Wang Y. (1995). Facilitation and depression at single central synapses. Neuron 14, 795–802. 10.1016/0896-6273(95)90223-6 7718241

[B36] Sumner C. J. Lopez-Poveda E. A. OMard L. P. Meddis R. (2002). A revised model of the inner-hair cell and auditory-nerve complex. J. Acoust. Soc. Am. 111, 2178–2188. 10.1121/1.1453451 12051437

[B37] Teich M. C. Lowen S. B. (1994). Fractal patterns in auditory nerve-spike trains. Eng. Med. Biol. Mag. IEEE 13, 197–202 10.1109/51.281678

[B38] Teich M. C. (1989). Fractal character of the auditory neural spike train. Biomed. Eng. IEEE Trans. 36, 150–160. 10.1109/10.16460 2921061

[B39] Tsodyks M. V. Markram H. (1997). The neural code between neocortical pyramidal neurons depends on neurotransmitter release probability. Proce. Natl. Acad. Sci. U.S.A. 94, 719–723. 10.1073/pnas.94.2.719 9012851 PMC19580

[B40] Van Steveninck R. D. R. Laughlin S. B. (1996). The rate of information transfer at graded-potential synapses. Nature 379, 642–645. 10.1038/379642a011517283

[B41] Wang X. J. (1999). Fast burst firing and short-term synaptic plasticity: a model of neocortical chattering neurons. Neuroscience 89, 347–362. 10.1016/S0306-4522(98)00315-7 10077318

[B42] Weber A. M. Wong F. K. Tufford A. R. Schlichter L. C. Matveev V. Stanley E. F. (2010). N-type Ca^2+^ channels carry the largest current: implications for nanodomains and transmitter release. Nat. Neurosci. 13, 1348–1350. 10.1038/nn.2657 20953196

[B43] Westerman L. A. Smith R. L. (1988). A diffusion model of the transient response of the cochlear inner hair cell synapse. J. Acoust. Soc. Am. 83, 2266–2276. 10.1121/1.396357 3411018

[B44] Zampini V. Johnson S. L. Franz C. Knipper M. Holley M. C. Magistretti J.. (2013). Burst activity and ultrafast activation kinetics of CaV1.3 Ca^2+^ channels support presynaptic activity in adult gerbil hair cell ribbon synapses. J. Physiol. 591, 3811–3820. 10.1113/jphysiol.2013.251272 23713031 PMC3764630

[B45] Zilany M. S. Carney L. H. (2010). Power-law dynamics in an auditory-nerve model can account for neural adaptation to sound-level statistics. J. Neurosci. 30, 10380–10390. 10.1523/JNEUROSCI.0647-10.2010 20685981 PMC2935089

[B46] Zilany M. S. Bruce I. C. Nelson P. C. Carney L. H. (2009). A phenomenological model of the synapse between the inner hair cell and auditory nerve: long-term adaptation with power-law dynamics. J. Acoust. Soc. Am. 126, 2390–2412. 10.1121/1.3238250 19894822 PMC2787068

[B47] Zilany M. S. Bruce I. C. Carney L. H. (2014). Updated parameters and expanded simulation options for a model of the auditory periphery. J. Acoust. Soc. Am. 135, 283–286. 10.1121/1.4837815 24437768 PMC3985897

